# NeoCLEAN: a multimodal strategy to enhance environmental cleaning in a resource-limited neonatal unit

**DOI:** 10.1186/s13756-021-00905-y

**Published:** 2021-02-12

**Authors:** A. Dramowski, M. Aucamp, A. Bekker, S. Pillay, K. Moloto, A. C. Whitelaw, M. F. Cotton, S. Coffin

**Affiliations:** 1 Department of Paediatrics and Child Health, Faculty of Medicine and Health Sciences, Stellenbosch University, PO Box 241, Cape Town, 8000 South Africa; 2 Unit for Infection Prevention and Control, Tygerberg Hospital, Cape Town, South Africa; 3 Division of Medical Microbiology, Department of Pathology, Faculty of Medicine and Health Sciences, Stellenbosch University and National Health Laboratory Service, Tygerberg Hospital, Cape Town, South Africa; 4 Division of Infectious Diseases, Children’s Hospital of Philadelphia and Department of Pediatrics, University of Pennsylvania Perelman School of Medicine, Philadelphia, USA

**Keywords:** Neonate, Bacterial colonization, Infection, Antimicrobial resistance, Cleaning, Africa

## Abstract

**Background:**

Contamination of the hospital environment contributes to neonatal bacterial colonization and infection. Cleaning of hospital surfaces and equipment is seldom audited in resource-limited settings.

**Methods:**

A quasi-experimental study was conducted to assess the impact of a multimodal cleaning intervention for surfaces and equipment in a 30-bed neonatal ward. The intervention included cleaning audits with feedback, cleaning checklists, in-room cleaning wipes and training of staff and mothers in cleaning methods. Cleaning adequacy was evaluated for 100 items (58 surfaces, 42 equipment) using quantitative bacterial surface cultures, adenosine triphosphate bioluminescence assays and fluorescent ultraviolet markers, performed at baseline (P1, October 2019), early intervention (P2, November 2019) and late intervention (P3, February 2020).

**Results:**

Environmental swabs (55/300; 18.3%) yielded growth of 78 potential neonatal pathogens with Enterococci, S*. marcescens, K. pneumoniae, S. aureus* and *A. baumannii* predominating*.* Highest aerobic colony counts were noted from moist surfaces such as sinks, milk kitchen surfaces, humidifiers and suction tubing. The proportion of surfaces and equipment exhibiting no bacterial growth increased between phases (P1 = 49%, P2 = 66%, P3 = 69%; *p* = 0.007). The proportion of surfaces and equipment meeting the ATP “cleanliness” threshold (< 200 relative light units) increased over time (P1 = 40%, P2 = 54%, P3 = 65%; *p* = 0.002), as did the UV marker removal rate (P1 = 23%, P2 = 71%, P3 = 74%; *p* < 0.001).

**Conclusion:**

Routine environmental cleaning of this neonatal ward was sub-optimal at baseline but improved significantly following a multimodal cleaning intervention. Involving mothers and nursing staff was key to achieving improved environmental and equipment cleaning in this resource-limited neonatal unit.

**Supplementary information:**

The online version contains supplementary material available at 10.1186/s13756-021-00905-y.

## Introduction

The hospital environment is a reservoir for bacterial pathogens and contributes substantially to the risk of healthcare-associated infections (HAIs) and outbreaks [1]. In the absence of cleaning, pathogens can persist in the hospital environmental for up to 30 months [2]. Despite being increasingly recognized as a contributor to HAIs, environmental contamination and hospital cleaning are seldom audited or reported from resource-limited settings [1].

Hospitalized neonates are at particularly high risk of developing HAIs owing to prematurity, use of invasive devices, broad-spectrum antibiotics and prolonged hospital stays [3]. For babies in most African maternity and neonatal units, the risk of developing HAIs is further exacerbated by overcrowding, understaffing, frequent sharing/re-use of equipment and limited implementation of infection prevention and control (IPC) programmes [4]. Poorly standardized cleaning practices, shortages of cleaning staff, contaminated water supplies [5, 6] and lack of training in decontamination of complex equipment, are additional factors that facilitate pathogen transmission [1, 2]. Interventions targeting these organizational, infrastructural and behavioral aspects of hospital cleaning practices that facilitate pathogen transmission, could reduce HAI rates in African maternity and neonatal units.

Studies modelling pathogen transmission in a neonatal unit using plant DNA demonstrate rapid and widespread environmental contamination [7]. From outbreak reports, many neonatal ward items are implicated as pathogen reservoirs including incubators, cot mattresses and toys [8–13]. Cross-transmission from these contaminated surfaces and equipment may transfer pathogens to neonates either directly or indirectly via contaminated hands. Inadequate environmental cleaning therefore promotes neonatal skin colonization with hospital pathogens and may lead to future invasive HAI episodes with newly acquired pathogens.

In many resource-limited settings, nurses are assigned responsibility for cleaning of the near-patient environment, particularly the cleaning of portable equipment [1, 14]. Additionally, sharing of equipment (e.g. saturation monitors, infusion pumps) is widespread owing to high occupancy rates and equipment shortages, with little time to clean and decontaminate equipment between patients. For this reason, cleaning interventions in resource-limited neonatal units should include a focus on nurse training in cleaning methods and equipment decontamination. Interventions to enhance cleaning adequacy in busy, understaffed African neonatal units should be low-cost, and simple to implement, measure and sustain. In addition, the potential role of mothers and neonatal caregivers in augmenting environmental cleaning in neonatal wards requires further study.

Environmental cleaning interventions are frequently employed during outbreaks [13] and for terminal cleaning to reduce the risk of pathogen transmission to the next room occupant [15]. In the last decade, hospital cleaning bundles [16] have become an effective way to improve routine cleaning and reduce HA-pathogen transmission [17, 18]. Key components of cleaning bundles include development of evidence-based policies, selection of appropriate cleaning products, staff education about cleaning, environmental cleaning monitoring and performance feedback [16, 18]. In contrast to the extensive experience with hospital cleaning bundles in high-income settings, there are few published reports on their use in low-resource setting and in particular, African country neonatal units [19, 20]. As hospital cleaning bundles are key tools for HAI prevention [17], research is needed to measure their impact on cleaning practices in resource-limited settings. Therefore, we assessed the impact of a multimodal intervention on the adequacy of surface and equipment cleaning in a South African neonatal ward.

## Methods

### Study design, setting and population

A prospective, quasi-experimental study was conducted in a 30-bed acute neonatal ward in Cape Town, South Africa to assess the impact of a multimodal intervention on surface and equipment cleaning. Tygerberg Hospital is a 1384-bed public teaching hospital, including maternity and neonatal services with 8000 high-risk deliveries (37% low birth weight) and approximately 2500 neonatal admissions annually. Despite being an upper middle-income country, South Africa has a high Gini coefficient indicating inequality; most patients utilizing this public hospital are indigent and the public hospital resources are more typical of a low-middle income country (LMIC) [21]. The 132-bed neonatal unit includes a 12-bed NICU, three high-dependency wards, and one kangaroo mother care ward, with mean occupancy rates exceeding 100%. The neonatal unit provides medical and surgical care for sick, preterm (< 37 weeks’ gestation) and/or low-birthweight (< 2500 g) neonates; prematurity, perinatal asphyxia and infection are the most common indications for admission. Given the extreme shortage of NICU beds, non-invasive ventilation (nCPAP and high-flow oxygen therapy) is used extensively in the high-dependency neonatal wards. The hospital’s on-site Unit for Infection Prevention and Control (UIPC) has one infection prevention nurse practitioner responsible for the maternity, paediatric and neonatal departments. Hand hygiene training and compliance monitoring is a major part of the IPC measures implemented in the neonatal unit. Parents are provided with hand hygiene training by the neonatal nurses and the UIPC conducts regular hand hygiene compliance monitoring. All staff and parents are required to perform hand hygiene at a dedicated handwash basin station at the entrance to the neonatal ward. In the patient cubicles, each neonatal cot/incubator/radiant warmer has an adjacent automated alcohol handrub dispenser or a plunger bottle with alcohol handrub.

### Cleaning policies and procedures prior to intervention

The UIPC presents quarterly training sessions to cleaning personnel and requires attendance of at least one training session per annum. Ad-hoc training sessions are given during outbreaks and to orientate newly-appointed cleaning staff. For routine cleaning of surfaces and equipment, water, household detergent and disposable cloths are used, and sometimes re-used. Spot cleaning is done immediately for spillage or gross contamination. Routine practices for the neonatal ward cleaning staff include daily wiping down of horizontal surfaces (work surfaces, tables, chairs and floors) and sinks plus twice daily emptying of waste bins. The cleaning personnel are responsible for cleaning of the milk kitchen, medication room, ward kitchen, toilet areas, mothers’ rooms and passages. The housekeeping supervisor uses a daily cleaning checklist to ensure that all the areas are cleaned. A color-coding system is used with separate cloths for patient, toilet, isolation and kitchen areas. The nursing staff are responsible for cleaning items in the patient zone i.e. all items touched directly or indirectly by the neonate or touched by the staff/mother while delivering care. These items included incubators, cots, bedside cabinets and clinical equipment; there was no system of monitoring the cleaning adequacy for these items prior to this study. Prior to implementation of the neoCLEAN intervention, the routine ward cleaning process did not specifically focus on high-touch areas. Apart from the cleaners’ daily environmental cleaning checklist for shared non-clinical areas (e.g. toilets, kitchens), there was neither a process to prioritize high-touch surfaces/equipment nor record the frequency of cleaning in clinical areas. Terminal cleaning is performed by nursing and cleaning staff after discharge or transfer of patients who were isolated and/or under transmission-based precautions using a checklist, followed by disinfection with 70% alcohol. Temperature probes for radiant warmers and saturation probes are disinfected with Clinell wipes between patients. Following patient discharge, incubators are fully disassembled for thorough cleaning with detergent and are then wiped down with 70% alcohol or Clinell wipes.

### Methods used to assess the adequacy of routine cleaning

The adequacy of routine cleaning was evaluated for 100 pre-specified items (58 surfaces and 42 equipment) using quantitative bacterial surface cultures, adenosine triphosphate (ATP) bioluminescence assays and fluorescent ultraviolet (UV) markers, performed at baseline (P1, October 2019), early intervention (P2, November 2019) and late intervention (P3, February 2020) phases. Surfaces and equipment (e.g. mattresses, cots, saturation monitors, infusion pumps) were swabbed using E-swabs in liquid transport medium (Sigma transwab); for flat surfaces a 10 × 10 cm swabbing template was used and for complex surfaces/equipment, a custom standard collection procedure was developed for each item. The swab was applied to the surface using a sweeping action in close parallel streaks while rotating the swab using a constant pressure. ATP relative light unit (RLU) readings were collected from the 100 specified surfaces and equipment using 3 M Clean-Trace swabs and a 3 M ATP meter. Ultraviolet (UV) disclosing lotion (GlitterBug Potion, Brevis Corp, Salt Lake City, UT) was applied to flat surfaces using a cotton bud in a circular motion in a 2 cm diameter; 100 UV marks were placed on the 100 specified surfaces and equipment items. The investigator returned after 24 h with a UV torch to count the proportion of markers that remained after 2 staff shift changes (i.e. after 2 opportunities for removal by routine cleaning).

### Metrics used to assess adequacy of cleaning

To assess the impact of the multi-modal intervention on cleaning adequacy we measured: (1) the proportion of the 100 pre-specified surfaces and equipment swabs with no bacterial growth and the proportion with growth of potential neonatal pathogens; (2) the proportion of ATP swab readings below an accepted threshold for cleanliness (< 200 relative light units [RLU]) [22] and (3) the proportion of UV marks removed following cleaning.

### Laboratory methods for the quantitative bacterial cultures

Surface and equipment swab tubes were vortexed for 30 s; 25 µL of the fluid was plated on to blood- and MacConkey agar plates and incubated at 37 °C for 48 h. Manual aerobic colony counts (ACC) were recorded for each plate and each unique colony was Gram stained. Gram-positive cocci were identified as *S. aureus*, coagulase negative staphylococci, enterococci, or streptococci through catalase testing, pyrrolidonyl aminopeptidase activity, and/or latex agglutination (Pastorex Staph-Plus; Bio-Rad, Redmond,WA). Gram-negative isolates were identified using the automated Vitek-2 system (BioMerieux, Marcy-l’Étoile, France). Agar plates with non-pathogens and skin flora (as defined by the Centers for Disease Control and Prevention) [23] were classified as “environmental/skin flora”; agar plates with neonatal pathogens e.g. *S. aureus* and Gram-negative bacilli, were classified as “potential pathogens.”

### NeoCLEAN multimodal intervention

The focus of the NeoCLEAN intervention was to improve cleaning of frequently-touched surfaces in the patient zone and areas associated with direct clinical care (medication trolley, supply and emergency trolleys, milk kitchen and hand washbasins). Other frequently-touched surfaces (e.g. telephones, computer keyboards) were included only if present in the clinical areas; toilet areas, sluice rooms, administrative offices and door handles to non-clinical areas were not included. The enhanced cleaning strategy focused mainly on nursing staff and mothers and was implemented in addition to the routine surface cleaning performed by the environmental cleaning staff. The key elements of the multi-modal NeoCLEAN intervention were training of nursing staff regarding surface/equipment cleaning, introduction of customized cleaning checklists, cleaning audits with staff feedback, use of in-room disinfectant wipes and involvement of neonates’ mothers with cleaning of the patient zone.

### Training of staff and mothers to clean the patient zone

Baseline training of nursing staff was conducted for approximately 1 h with 30 neonatal ward nursing staff (in 4 sessions to include day and night shifts). The training reinforced why enhanced cleaning is needed for neonatal wards to maximize “buy-in” and cooperation from staff with the intervention. New cleaning standards were set and cleaning procedures were clarified by discussion e.g. what needed to be cleaned, how, when, how often, and by whom. Clearly delineating the roles and cleaning responsibilities of cleaning staff, nursing staff and mothers was a key task prior to implementing the intervention. Each nurse working in the clinical rooms took responsibility for teaching the mothers of newly admitted neonates to perform daily cleaning of their babies’ cots, incubators and bedside cabinets.

### Development of cleaning checklists

**Fig. 1:**
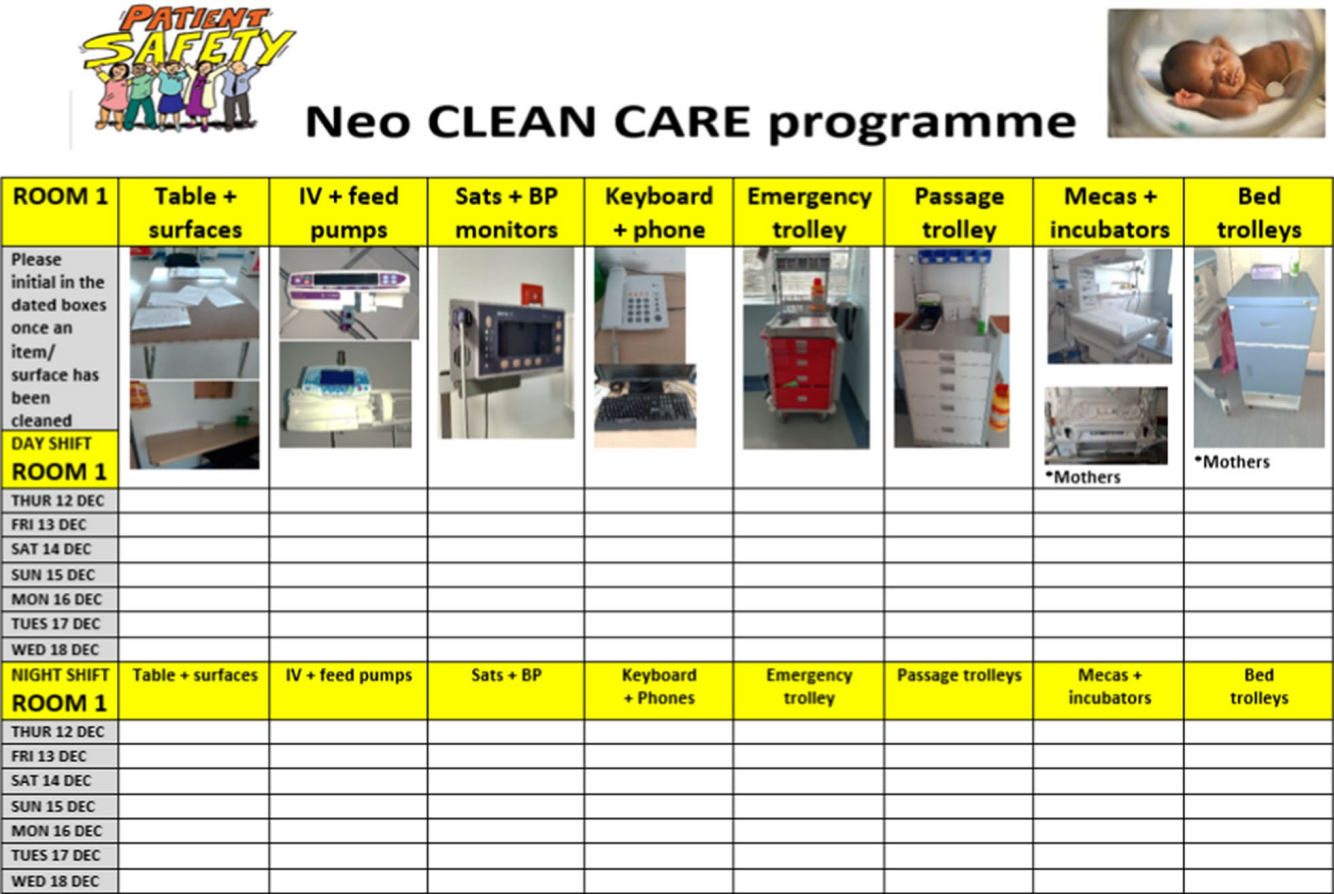
Cleaning checklist for a neonatal patient cubicle

Clinical observation was conducted in each area to determine the most frequently touched surfaces. For each area targeted (6 patient cohort rooms, a medication preparation area and the milk kitchen), a specific checklist was designed based on the surfaces/equipment present. The checklist included color photographs of the items to be cleaned with space for day and night shift staff to sign after completing cleaning (Fig. 1). The checklists were prominently displayed in each room, acting as a visual reminder to prompt cleaning. A new blank checklist was placed in each room every week. Frequent reminders had to be given initially to ensure staff compliance with completing the checklist.

### Introduction of disinfectant wipes

Commercially available pre-packaged hospital disinfectant wipes (Clinell TM Universal wipes) were placed in an easily accessible position in each clinical room, the medication preparation area and the milk kitchen. The Clinell wipes (active ingredients benzalkonium chloride, didecyldimonium chloride and phenoxyethanol) utilize a one-step cleaning and disinfection process which reduces the cleaning time. When Clinell wipes were out of stock, staff were trained to use alternative cleaning agents e.g. cleaning with liquid detergent and water and drying, followed by disinfection with 70% alcohol spray.

### Implementation of cleaning audits with staff feedback

The multimodal intervention was implemented from 1 November 2019 to 28 February 2020. Three formal assessments of neonatal ward cleaning adequacy were performed at baseline (P1, October 2019), early intervention (P2, November 2019) and late intervention (P3, February 2020). Following each phase, verbal feedback and encouragement was provided to the neonatal nurses and the assessment results were prominently displayed on the ward’s infection prevention noticeboard. Intermittent, informal cleaning audits with feedback were conducted over the 5-month study period. These informal audits used UV marks to provide visual feedback to staff on frequently missed surfaces when cleaning. The audits were also an opportunity to hear how the nursing staff perceived the cleaning intervention, identify obstacles to cleaning and record suggestions for programme improvement.

### Data collection, statistical analysis and study approvals

Data for each assessment phase were collected on a report form for the 100 surfaces/equipment items for bacterial cultures, UV gel markers and ATP swabs then entered into a REDCap database. Proportions were calculated for each of the test metrics at each study phase. Continuous and categorical variables were compared using the Kruskal–Wallis test and the *X*2 test, respectively. A *p* value of < 0.05 was considered statistically significant. Stata Statistical Software version 13.0 IC (College Station, TX: StataCorp LP) was used for analysis. At the informal ward cleaning audits, the investigators collected and manually recorded verbatim feedback from nursing staff and mothers. The Stellenbosch University Health Research Ethics Committee and the Tygerberg Hospital management approved the study protocol (N18/07/068).

## Results

Over the 3 assessment phases, 55/300 (18.3%) surface and equipment swabs yielded growth of 78 potential neonatal pathogens, most commonly, Enterococci, S*. marcescens, K. pneumoniae, S. aureus* and *A. baumannii.* (Table 1)*.* The median aerobic colony count (ACC) from swabs with any bacterial growth (n = 116) was 6 (IQR 1–57). Highest ACC and a predominance of Gram-negative pathogens were noted from moist surfaces and equipment e.g. sinks, milk kitchen surfaces, humidifiers, and suction tubing.

**Table 1:** Potential neonatal pathogens isolated from swabs of surfaces and equipment

	Overall^a^ (P1, P2, P3)	P1 (baseline)	P2 (early intervention)	P3 (late intervention)
Proportion of swabs with growth of potential neonatal pathogens	55/300 (18.3%)	22 (22%)	19 (19%)	14 (14%)
Total pathogens isolated^b^	78	32	28	18
*Enterococcus species*	11	2	6	3
*Serratia species*	10	2	6	2
*Acinetobacter species*	6	3	1	2
*Klebsiella species*	7	1	4	2
*S. aureus*	7	3	3	1
Other potential bacterial pathogens^c^	37	21	8	8

^a^During the 3 study phases (P1, P2, P3) 55/300 swabs (18.3%) yielded growth of 75 potential neonatal pathogens

^b^Some swabs isolated more than one potential neonatal pathogen

^c^Other potential bacterial pathogens isolated were: Pseudomonas spp (n = 6), Aeromonas spp (n = 5), Enterobacter spp (n = 4), Pantoea spp (n = 3), Pasteurella spp (n = 3), Sphingomonas spp (n = 2), *E.coli* (n = 2), Group B Streptococcus (n = 2), *E. meningosepticum* (n = 2), *U. urealyticum*, *S. pneumoniae*, *G. morbillorum*, *B. cepaciae*, *S. maltophilia*, *S. sonnei,* Ewingella spp and Nocardia spp (n = 1 each)

**Fig. 2:**
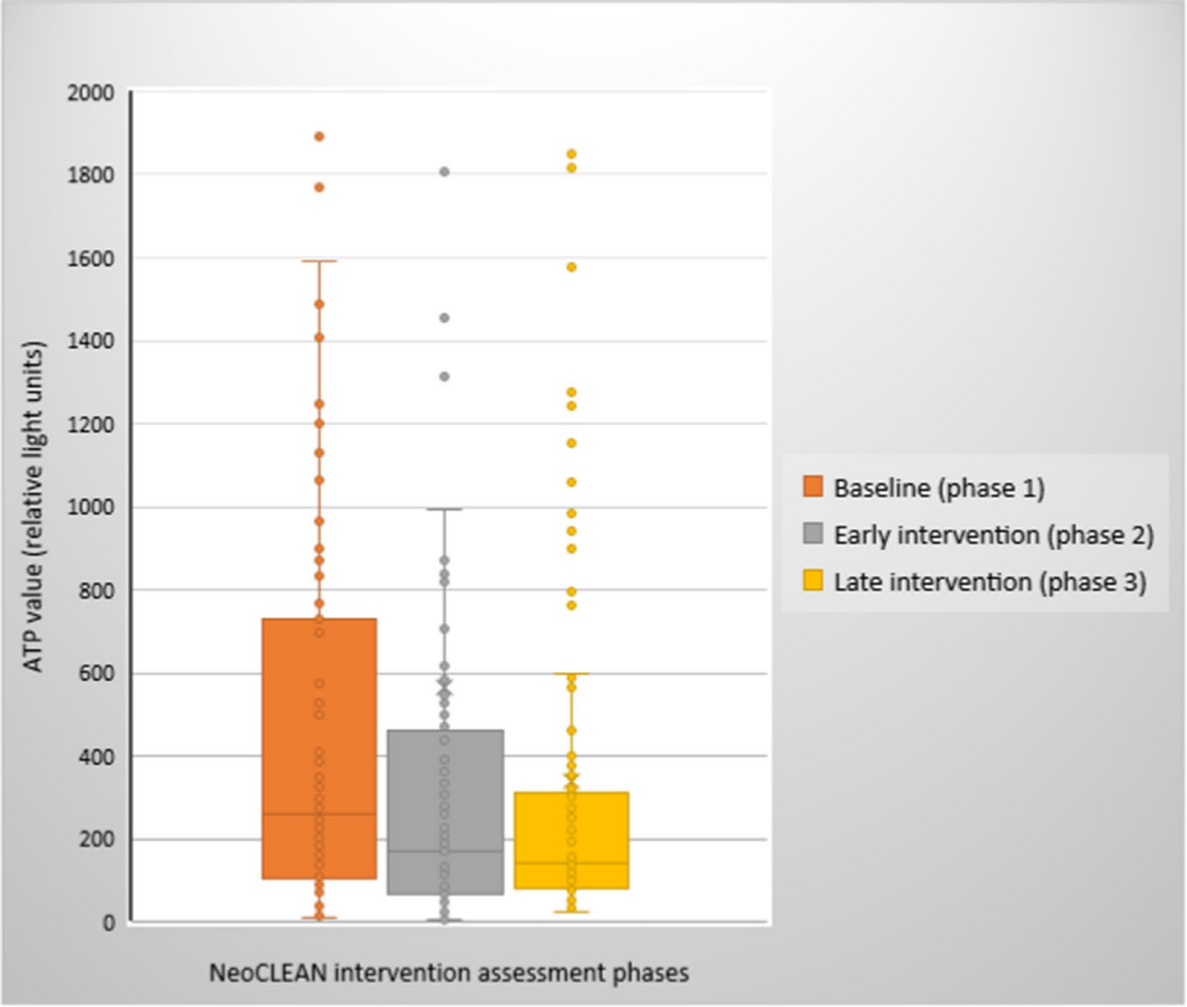
Comparison of ATP surface and equipment contamination between intervention phases. The median ATP swab values (in RLU) for surfaces and equipment declined significantly between assessment phases (Phase 1 = 261 [IQR 108–731], Phase 2 = 171 [IQR 70–456], Phase 3 = 143 [IQR 79–315]; *p* = 0.007)

The proportion of surfaces and equipment exhibiting no bacterial growth increased between assessment phases (P1 = 49%, P2 = 66%, P3 = 69%; *p* = 0.007). The proportion of surfaces yielding potential neonatal pathogens decreased between assessment phases but did not reach statistical significance (P1 = 22%, P2 = 19%, P3 = 14%; *p* = 0.336). The proportion of surfaces and equipment meeting the “cleanliness” threshold (< 200 RLU) increased over time (P1 = 40%, P2 = 54%, P3 = 65%; *p* = 0.002) (Table 2). Similarly, the median ATP RLU values for surfaces and equipment declined significantly between assessment phases (P1 = 261 [IQR 108–731], P2 = 171 [IQR 70–456], P3 = 143 [IQR 79–315]; *p* = 0.007) (Fig. 2). There was no difference in the median ATP RLU from surfaces compared to equipment over the 3 assessment phases (207 RLU [IQR 94–518] versus 163 RLU [IQR 62–464]; *p* = 0.116).

**Table 2:** Impact of the neoCLEAN multimodal intervention on the adequacy of routine environmental cleaning

Metric to assess the adequacy of routine cleaning	Phase of the neoCLEAN multimodal intervention			*p* value
	P1 (baseline) (%)	P2 (early intervention) (%)	P3 (late intervention) (%)	
Proportion of swabs^a^ with no bacterial growth	49	66	69	*p* = 0.007
Proportion of swabs^a^ yielding potential pathogens	22	19	14	*p* = 0.336
Proportion of UV gel markers removed	23	71	74	*p* < 0.001
Proportion of ATP swab readings < 200 relative light units (RLU)	40	54	65	*p* = 0.002

^a^Swabs from surface and equipment items evaluated (n = 100) included: stethoscopes (n = 4); flat surfaces of tables and workbenches (n = 8); computer keyboards (n = 2); telephones (n = 2); sinks (n = 4); medication trolley (n = 1); cots (n = 5); mecas (n = 5); incubators (n = 10); incubator/cot mattresses (n = 5); medical notes folders (n = 5); baby scale (n = 1); intravenous fluid infusion pumps (n = 5); saturation monitors (n = 5); bedside trolleys (n = 5); CPAP machines (n = 2); nasal cannula oxygen humidifier water (n = 7); suction tubing/bottles (n = 4); laryngoscope blade (n = 1); haemoglobinometer (n = 1); glucometer (n = 1); thermometers (n = 2); scissors (n = 2); milk fridges (n = 2); paper towel dispensers (n = 4); staff mobile phones (n = 5)

The rate of UV mark removal following cleaning increased significantly over time (P1 = 23%, P2 = 71%, P3 = 74%; *p* < 0.001) (Table 2). Flat surfaces, as compared to equipment, had a higher rate of UV mark removal over the 3 phases (132/174 [75.9%] vs 36/126 [28.6%]; *p* < 0.001). Items with the lowest UV mark removal rate (i.e. frequently missed) were saturation monitors, intravenous infusion pumps and enteral feeding pumps.

The neonatal bloodstream infection rate for the ward declined following implementation of the NeoCLEAN intervention, from 6.7/1000 patient days in the 4 months preceding the intervention (July–October 2019) to 3.9/1000 patient days during the intervention period (November 2019 – February 2020) (*p* = 0.166).

The NeoCLEAN intervention was well received with most nurses and mothers reporting a positive attitude to cleaning and an understanding that cleaning was an important contributor to patient safety.

- Mother 1: ‘I can help my baby to be safe from infection by cleaning properly.’
- Nurse 3: ‘I feel we are working more like a team now…how I clean today on my shift will make a difference to neonatal care.’

Although frequent reminders to staff were required after the initial NeoCLEAN training session, nurses soon improved their compliance with 12-hourly checklist completion. Nurses reported that the photographic images of the items requiring cleaning on the checklist helped them in prioritizing items and surfaces to clean.

- Nurse 1: ‘Sometimes we are very busy and don’t clean immediately when coming on shift, but the checklist helps us keep track of what we must still do, in between feeding babies and taking the observations.’
- Nurse 2: ‘I like the pictures on the checklist—it helps me remember what I must still clean.’

Nurses took the lead in training mothers on how to clean their baby’s immediate environment, but remained willing to ensure that cleaning was thorough, even when mothers were absent, unable or unwilling to partake in cleaning efforts.

- Nurse 4: ‘Some mothers are very helpful and want to clean their baby’s space. For those that don’t want to, we do it for them.’

As implementation of the intervention with regular cleaning audits continued, nursing staff and mothers became familiar with the UV gel marker assessment method and which surfaces /equipment required cleaning.

- Mother 2 to IPC practitioner: ‘I know what you are coming to do in this room with your light of truth!’

## Discussion

Given the upward trends in population growth, urbanization and in-hospital births in African countries, admission volumes in busy maternity and neonatal units will likely increase further. The risk of developing neonatal HAI is also likely to increase with higher occupancy rates and expanded access to neonatal interventions such as surfactant therapy and non-invasive respiratory support. Enhanced hospital cleaning has an important role to play in reducing neonatal HAI risk in Africa. Ours is the first study to evaluate the potential of environmental cleaning as a standalone HAI prevention intervention, with only one prior study including cleaning in a neonatal infection prevention bundle [20]. Furthermore, the key contribution of hospital cleaners and cleaning practices to patient safety is underappreciated in many LMIC settings [24], despite the substantial risk of pathogen transmission from environmental reservoirs [4–6].

In this paper, we report the impact of a multimodal intervention (neoCLEAN) on the adequacy of surface and equipment cleaning in a South African neonatal ward using bacterial cultures, ATP assays and UV marker removal rates. At the pre-intervention (baseline) cleaning assessment, almost 1 in 4 surfaces and equipment swabbed yielded growth of potential neonatal pathogens. Of the bacteria isolated, the 5 most frequent species identified were also the leading pathogens causing HA-bloodstream infection at this neonatal unit (Enterococci, S*. marcescens, K. pneumoniae, S. aureus, A. baumannii)* [25, 26], supporting a link between environmental reservoirs and neonatal HAI. Although not statistically significant, we also demonstrated a decline in the neonatal bloodstream infection rate following implementation of the NeoCLEAN intervention.

Microbiological analysis of surface swabs from a Moroccan neonatal unit also showed a predominance of Gram-negative pathogens, particularly Enterobacterales, *Acinetobacter baumannii* and *Pseudomonas aeruginosa*, many of which were multi-drug resistant [19]. As in the present study, levels of bacterial growth from surface swabs were generally low [19]. The Moroccan NICU identified fixed equipment (52%), sinks (15%), incubators/cribs (15%), door handles (11%) and computers (8%) as the most frequently contaminated items. However, in our neonatal ward, moist surfaces and equipment, including sinks, milk kitchen surfaces, humidifiers, and suction tubing had the highest colony counts and pathogen contamination rates, emphasizing the importance of decontaminating wet areas. In a study of Malawian obstetric units, high rates of Gram-negative contamination were identified from samples of the hospital water supply (23%) [6], highlighting the potential contribution of water to environmental pathogen reservoirs.

During the NeoCLEAN intervention, the use of UV gel markers to provide real-time feedback to nursing staff was particularly useful for reinforcing cleaning of equipment items that were frequently missed e.g. saturation monitors and infusion pumps. Previous studies have noted that surface cleaning and disinfection tasks are prone to "human errors" such as failure to clean key items [27]. A major benefit of using disposable surface wipes were that these are waterless (avoiding external contamination) and single use (avoiding sharing of cleaning cloths with potential to transfer pathogens) [27].

Prior studies of hospital cleaning in resource-limited settings identified lack of training, low societal value attached to cleaning, and disempowerment of cleaning staff, as major factors that perpetuate poor cleaning performance [5, 24]. In a survey of healthcare facilities in India, Bangladesh, The Gambia and Zanzibar [24], few gave formal training to their cleaning staff and the role of cleaning was generally not perceived as important. As African obstetric and neonatal units strive to attain the Sustainable Development Goals for maternal and newborn health and survival and reduce the spread of antimicrobial resistant pathogens, programmes to enhance facility cleaning should be promoted.

The neoCLEAN intervention incorporated several elements described in effective cleaning bundles including education, audit with feedback and checklists [15, 17]. A key element in the NeoCLEAN intervention was use of a single-step cleaning/disinfectant wipe, available at the point-of-use in every clinical room. These may not be widely available in resource-limited settings owing to cost, but are likely to be cost-effective given the time saved for busy neonatal nurses. Training and empowerment of neonatal ward mothers to perform twice daily cleaning of their infant’s crib/incubator and bedside cabinet, was another important element that reduced the cleaning workload for nurses. Challenges encountered during the 5-month study included a month where the disinfectant wipes were unavailable and alternative cleaning agents had to be provided.

A challenge for the nursing managers was lack of time for cleaning audits and feedback, although they emphasized the importance of completing the cleaning checklists at each shift change. Initially staff needed frequent prompting to ensure compliance with cleaning and checklist completion, but staff later embraced the new cleaning routine as a daily habit. The key factors contributing to changes in staff cleaning behaviors were: (1) convincing staff that enhanced cleaning was needed; (2) defining which items to clean; (3) making cleaning faster and easier with empowerment of mothers to assist; (4) repeated verbal reminders and positive feedback to reinforce desired behaviors and (5) prominently displaying checklists as visual reminders for cleaning.

Limitations of this study include the single implementation site and the short period of follow-up of 5 months. We did not classify coagulase negative staphylococci as potential pathogens, as they are extremely infrequent pathogens in our setting [26]. Strengths of this study are the use of three different assessment methods to evaluate the adequacy of cleaning and the implementation of a novel cleaning bundle in a high-risk population: hospitalized neonates in a resource-limited unit. Given the extremely limited evidence base for implementation of cleaning bundles in African neonatal units, more research is needed to identify the most efficacious, feasible and cost-effective interventions to enhance routine cleaning. Future studies should ideally include long-term follow-up to assess the sustainability of improvements in neonatal ward cleaning following cessation of the intervention.

## Conclusions

Environmental cleaning in this neonatal ward was sub-optimal at baseline but improved significantly following a multimodal cleaning intervention. Involving mothers and nursing staff was key to sustaining improvement in environmental cleaning in this resource-limited neonatal unit.

## Supplementary Information

**Additional file 1**. List of the 100 Items and surfaces sampled in the neonatal unit.

## Data Availability

The datasets generated during and/or analysed during the current study are available from the corresponding author on reasonable request.

## Abbreviations

ACC: Aerobic colony count
ATP: Adenosine triphosphate
HAI: Healthcare-associated infection
IPC: Infection prevention and control
LMIC: Low-to-middle income countries
nCPAP: Nasal cannula positive airways pressure
NICU: Neonatal intensive care unit
P: Phase
RLU: Relative light units
spp: Species
UIPC: Unit for Infection Prevention and Control
UV: Ultraviolet
