# Supplementary material for: NeoCLEAN: a multimodal strategy to enhance environmental cleaning in a resource-limited neonatal unit
Source: Antimicrob Resist Infect Control. 2021 Feb 12;10:35. doi: 10.1186/s13756-021-00905-y (PMC7881651; doi:10.1186/s13756-021-00905-y)
Supplement: Supplementary file 1 — Additional file 1. List of the 100 Items and surfaces sampled in the neonatal unit. [file 13756_2021_905_MOESM1_ESM.docx]

| **Supplementary file: Items and surfaces sampled in the neonatal unit** | | | |
| --- | --- | --- | --- |
| **Swab Number** | **Description** | **Swab Number** | **Description** |
| 1 | Stethoscope A | 51 | Infusion pump room 1 |
| 2 | Stethoscope B | 52 | Infusion pump room 2 |
| 3 | Stethoscope C | 53 | Infusion pump room 3 |
| 4 | Stethoscope D | 54 | Infusion pump room 4 |
| 5 | Room 1 flat surface | 55 | Infusion pump room 5 |
| 6 | Room 2 flat surface | 56 | Saturation monitor room 1 |
| 7 | Room 3 flat surface | 57 | Saturation monitor room 2 |
| 8 | Room 4 flat surface | 58 | Saturation monitor room 3 |
| 9 | Room 5 flat surface | 59 | Saturation monitor room 4 |
| 10 | Room 6 flat surface | 60 | Saturation monitor room 5 |
| 11 | Computer keyboard A | 61 | Bedside trolley 1 |
| 12 | Computer keyboard B | 62 | Bedside trolley 2 |
| 13 | Ward telephone room 1 | 63 | Bedside trolley 3 |
| 14 | Ward telephone room 2 | 64 | Bedside trolley 4 |
| 15 | Communal handwash basin | 65 | Bedside trolley 5 |
| 16 | Handwash basin med prep | 66 | Oxygen humidifier bottle 1 |
| 17 | Medication trolley | 67 | Oxygen humidifier bottle 2 |
| 18 | Cot room 2 | 68 | Oxygen humidifier bottle 3 |
| 19 | Cot room 3 | 69 | Oxygen humidifier bottle 4 |
| 20 | Cot room 4 | 70 | Oxygen humidifier bottle 5 |
| 21 | Radiant warmer 1 | 71 | Oxygen humidifier bottle 7 |
| 22 | Radiant warmer 2 | 72 | Oxygen humidifier bottle 7 |
| 23 | Incubator 1 | 73 | CPAP circuit 1 |
| 24 | Incubator 2 | 74 | CPAP circuit 2 |
| 25 | Incubator 3 | 75 | CPAP circuit 3 |
| 26 | Incubator 4 | 76 | CPAP circuit 4 |
| 27 | Incubator 5 | 77 | Suction bottle room 1 |
| 28 | Incubator 6 | 78 | Suction bottle room 2 |
| 29 | Incubator 7 | 79 | Suction bottle room 3 |
| 30 | Incubator 8 | 80 | Laryngoscope blade |
| 31 | Mattress 1 | 81 | Silicon facemask |
| 32 | Mattress 2 | 82 | Haemoglobinometer |
| 33 | Mattress 3 | 83 | Glucometer |
| 34 | Mattress 4 | 84 | Thermometer 1 |
| 35 | Mattress 5 | 85 | Thermometer 2 |
| 36 | Medical folder 1 | 86 | Nurses scissors 1 |
| 37 | Medical folder 2 | 87 | Nurses scissors 1 |
| 38 | Medical folder 3 | 88 | Milk kitchen surface 1 |
| 39 | Medical folder 4 | 89 | Milk kitchen surface 2 |
| 40 | Medical folder 5 | 90 | Breastmilk fridge 1 |
| 41 | Handwash basin room 2 | 91 | Breastmilk fridge 2 |
| 42 | Milk kitchen sink | 92 | Paper towel dispenser 1 |
| 43 | Baby weighing scale | 93 | Paper towel dispenser 2 |
| 44 | Incubator 9 | 94 | Paper towel dispenser 3 |
| 45 | Incubator 10 | 95 | Paper towel dispenser 4 |
| 46 | Cot room 5 | 96 | Staff mobile phone 1 |
| 47 | Cot room 6 | 97 | Staff mobile phone 2 |
| 48 | Radiant warmer 3 | 98 | Staff mobile phone 3 |
| 49 | Radiant warmer 4 | 99 | Staff mobile phone 4 |
| 50 | Radiant warmer 5 | 100 | Staff mobile phone 5 |
